# A Double-Edged Sword: Quality and Credibility of Colon Cancer Screening Content on YouTube

**DOI:** 10.7759/cureus.54929

**Published:** 2024-02-26

**Authors:** Rajmohan Rammohan, Sai Greeshma Magam, Melvin Joy, Wing Hang Lau, Abhishek Tadikonda, Dilman Natt, Sai Reshma Magam, Leeza Pannikodu, Jiten Desai, Rucha Jiyani, Saher Sheikh, Susan Bunting, Prachi Anand, Krishnaiyer Subramani, Nausheer Khan, Paul Mustacchia

**Affiliations:** 1 Gastroenterology, Nassau University Medical Center, East Meadow, USA; 2 Internal Medicine, Nassau University Medical Center, East Meadow, USA; 3 Rheumatology, Nassau University Medical Center, East Meadow, USA; 4 Gastroenterology and Hepatology, Nassau University Medical Center, East Meadow, USA

**Keywords:** colon cancer awareness, academic and private, youtube education, colon cancer prevention, youtube videos

## Abstract

Introduction

Colorectal cancer (CRC) remains a significant public health challenge globally, with its pathogenesis involving the transformation of benign adenomas into malignant carcinomas. Despite advancements in screening and early detection significantly improving outcomes, the rise of digital platforms like YouTube for disseminating health information presents new challenges. Concerns over the accuracy and reliability of content underline the necessity for rigorous evaluation of these digital health education tools.

Methods

Our study was conducted at Nassau University Medical Center, East Meadow, New York. We meticulously analyzed YouTube videos on "colon cancer screening awareness," employing strict selection criteria to ensure both relevance and quality, focusing on English-language content with pertinent audio. Videos were evaluated for their quantitative and qualitative attributes-views, subscriber counts, likes/dislikes, comments, and content type, classifying them as scholarly or personal. We assessed video credibility through scientific accuracy using the DISCERN instrument, Global Quality Score (GQS), and Patient Education Materials Assessment Tool (PEMAT), ensuring consistency in quality and reliability evaluation among seven researchers via the intraclass correlation coefficient. These tools - DISCERN for assessing reliability and quality, GQS for evaluating overall quality, and PEMAT for understandability and actionability - facilitated a comprehensive evaluation framework. Our analysis, leveraging descriptive and inferential statistics, scrutinized differences in content quality between academic and private institutions, employing t-tests to identify statistically significant disparities. The study utilized Microsoft Excel (version 16.73, Microsoft Corporation, Redmond, Washington, United States) and IBM SPSS Statistics for Windows, version 29.0 (released 2022; IBM Corp., Armonk, New York, United States). for robust data processing and analysis, confirming the educational value and trustworthiness of the examined YouTube content.

Results

Our study of 156 YouTube videos on educational content, split between academic (68 videos) and private sources (88 videos), revealed significant quality differences. Using the DISCERN, PEMAT, and GQS metrics, academic videos consistently outperformed private ones, with significant margins: DISCERN (54.61 vs. 34.76), PEMAT (3.02 vs. 2.11), and GQS (3.90 vs. 2.02), supported by low p-values indicating a statistically significant superiority. These findings suggest that the source of content-academic versus private-plays a crucial role in determining the quality and reliability of educational materials on platforms like YouTube, highlighting the academic sector's commitment to higher educational standards.

Conclusion

The study emphasizes the critical role of credible sources in enhancing the quality of health education content on YouTube, particularly concerning CRC screening. The superiority of academic institutions in providing high-quality content suggests a need for viewers to critically assess the source of information. It also calls for enhanced regulatory oversight and measures to ensure the accuracy and reliability of health information online.

## Introduction

Colorectal cancer (CRC) is a major global health concern, ranking third in prevalence and second in mortality among cancers, as highlighted by the Global Cancer Observatory (GLOBOCAN) 2020 [1]. The pathogenesis of CRC involves the disruption of normal cell cycle processes in epithelial cells, leading to uncontrolled growth and the formation of benign growths known as adenomas. These adenomas have the potential to develop into malignant adenocarcinomas and eventually metastasize, primarily following the adenoma-carcinoma sequence [2]. Various factors contribute to the risk of CRC, including lifestyle choices, such as diet and smoking, and unchangeable factors, like age, ethnicity, and genetic predisposition.

Screening for CRC is a vital preventive measure, significantly impacting patient outcomes through early detection and treatment. Studies have demonstrated that adenomas, the precursors to CRC, can take over a decade to transform into carcinomas, offering a window for intervention [3]. In response to the rising incidence of early-onset CRC, particularly in individuals aged 45-49, recent guidelines have lowered the recommended age for starting CRC screening to 45 years [4]. This proactive approach aims to catch and treat CRC at its earliest stages, thereby reducing mortality rates [5,6].

In today's digital era, YouTube has emerged as a significant platform for disseminating health information. Healthcare professionals increasingly use this medium to educate the public and improve health literacy. However, this trend is not without its challenges. There is growing concern among health experts about the accuracy and reliability of health information shared on YouTube. The lack of regulatory oversight on the platform raises the risk of misinformation, which can have serious implications for public health understanding and practices [7].

This paper was presented at the American College of Gastroenterology Conference in Vancouver, Canada, in 2023.

## Materials and methods

Data collection

Our research was conducted at Nassau University Medical Center, East Meadow, New York. We meticulously searched YouTube for content related to "colon cancer screening awareness," applying stringent criteria to ensure relevance and quality. Our initial step was to filter out videos that did not meet our language requirement (English only), were unrelated to colon cancer screening, or were devoid of audio. We then analyzed these videos based on several quantitative and qualitative factors, including the number of views, subscriber count, likes, dislikes, comments, and the nature of the content, categorizing them as either scholarly or personal. The core of our assessment involved evaluating the credibility of each video, focusing on its scientific accuracy. To measure the overall quality of the content, we employed three distinct tools, namely, the DISCERN instrument [8], the GQS [9], and the PEMAT [10], which were widely used to measure the quality and reliability of the videos in the studies. To validate our evaluation process, we relied on the intraclass correlation coefficient, ensuring a reliable consensus among seven researchers on the application of DISCERN, GQS, and PEMAT scores, thereby establishing a comprehensive framework for determining the educational value and trustworthiness of the videos in question.

DISCERN Scale

The DISCERN instrument, originally crafted for assessing the quality and reliability of written health information, extends its applicability to YouTube videos, offering a structured approach to evaluate content across three main areas: reliability, quality of treatment information, and overall quality. Each aspect is rated on a scale from 1 to 5, reflecting the clarity, relevance, and balance of the information presented, as shown in Table 1. This method aids in systematically determining the credibility and educational value of health-related videos, guiding viewers toward informed and trustworthy resources [8].

**Table 1:** DISCERN score Scoring is done on a scale from 0 to 5, where 0 is the lowest score, indicating poor performance, and 5 is the highest, indicating excellent performance in the evaluated criteria

Questions	
Are the aims clear?	(0-5)
Does it achieve its aims?	(0-5)
Is it relevant?	(0-5)
Is it clear what sources of information were used to compile the publication (other than the author or producer)?	(0-5)
Is it clear when the information used or reported in the publication was produced?	(0-5)
Is it balanced and unbiased?	(0-5)
Does it provide details of additional sources of support and information?	(0-5)
Does it refer to areas of uncertainty?	(0-5)
Does it describe how each treatment works?	(0-5)
Does it describe the benefits of each treatment?	(0-5)
Does it describe the risks of each treatment?	(0-5)
Does it describe what would happen if no treatment is used?	(0-5)
Does it describe how the treatment choices affect the overall quality of life?	(0-5)
Is it clear that there may be more than one possible treatment choice?	(0-5)
Does it provide support for shared decision-making?	(0-5)
Based on the answers to all of the above questions, rate the overall quality of the publication as a source of information about treatment choices	(0-5)

GQS

The GQS evaluates the overall quality of health-related YouTube videos on a 1 to 5 scale, assessing their accuracy, depth, clarity, and usefulness. This score helps differentiate high-quality, informative content from less reliable sources, emphasizing the importance of credible, evidence-based information in health education [9].

PEMAT Score

The PEMAT score, used to assess the clarity and practicality of patient education materials, focuses on two key aspects: understandability and actionability. Understandability assesses the ease with which the target audience grasps the provided information. The criteria include message clarity, simple language use, content organization, the inclusion of visual aids, and the pacing of information delivery. Actionability evaluates how well the material instructs viewers on subsequent steps. This involves checking if the material clearly outlines the actions to be taken post-viewing, with clear instructions or demonstrations as needed. Scoring is done on a scale from 1 to 5, where 1 is the lowest score, indicating poor performance, and 5 is the highest, indicating excellent performance in the evaluated criteria [10].

Analysis 

Descriptive Statistics

In the analysis of YouTube content quality across academic and private institutions, we employed a comprehensive statistical approach to evaluate differences in content quality metrics: DISCERN, PEMAT, and GQS. Initially, the dataset was segmented based on the type of institution (academic vs. private), ensuring a clear comparison between the two groups. Descriptive statistics were calculated for each group and metric, providing insights into central tendencies (mean) and variability (standard deviation) within the data. This step was crucial for understanding the distribution and central characteristics of the scores across both institution types.

Hypothesis Testing

Subsequently, inferential statistical methods were utilized to assess the significance of the observed differences between the academic and private groups. Specifically, independent samples t-tests were conducted for each metric. These tests are designed to compare the means of two independent groups under the assumption of normal distribution and unequal variances (hence, the use of Welch's t-test variation). The p-values obtained from these t-tests served as the basis for determining statistical significance, with lower values indicating stronger evidence against the null hypothesis of no difference between group means.

This methodological framework, combining descriptive and inferential statistics, provided a robust analysis of the dataset. It allowed us to quantify the extent of differences in content quality metrics between academic and private institutions and to ascertain the statistical significance of these differences, thereby offering a solid foundation for conclusions drawn from the data.

Data processing software

For the statistical analysis in this study, data processing and calculations were conducted using Microsoft Excel (Version 16.73, Microsoft Corporation, Redmond, Washington, United States) and IBM SPSS Statistics for Windows, version 29.0 (released 2022; IBM Corp., Armonk, New York, United States). The analytical approach involved performing repeated unpaired two-sample t-tests to compare the means between the two groups under examination. The significance of the results was determined based on a two-tailed p-value approach, with a threshold set at less than 0.05 to denote statistical significance. This methodology ensured a robust and reliable data analysis, providing a solid foundation for the study's conclusions.

Ethics approval

This research evaluated the public contributions and perceptions regarding the quality of YouTube, thus deeming the approval of an ethics committee non-essential.

## Results

**Figure 1:**
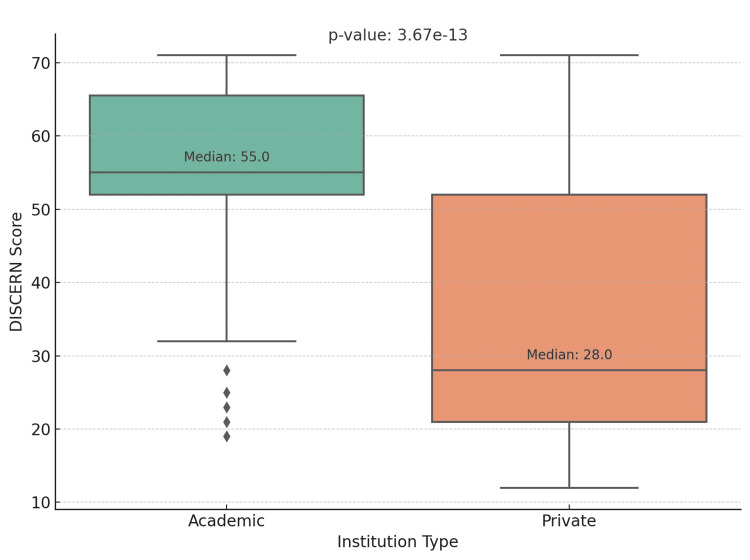
DISCERN score DISCERN score (1-80): 1 being the lowest and 80 being the highest

**Figure 2:**
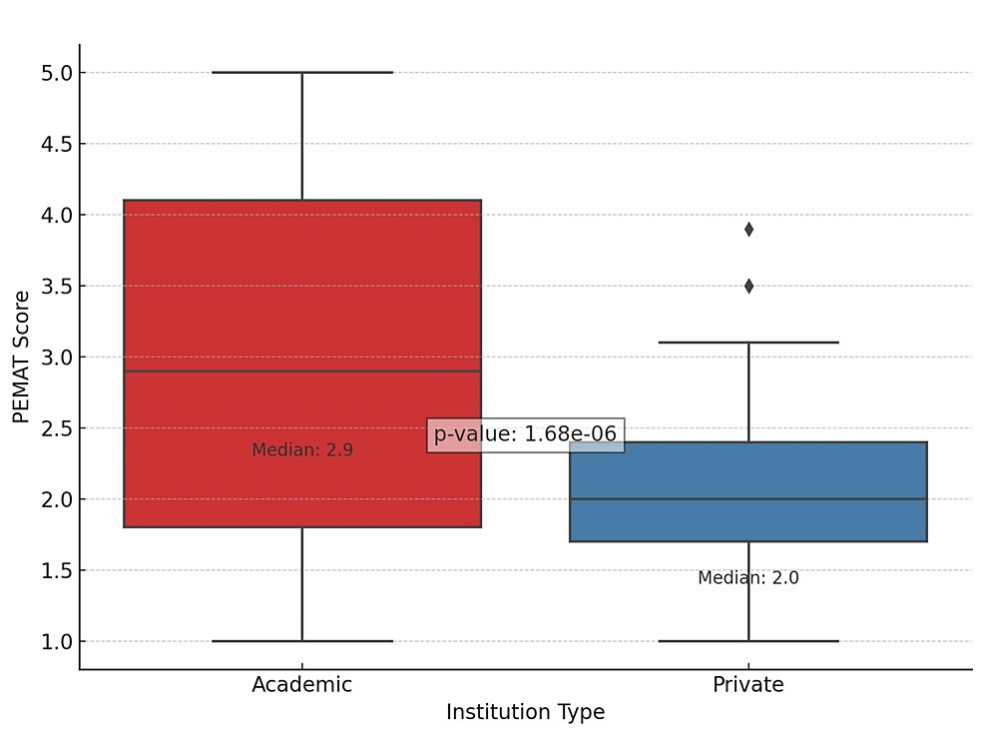
PEMAT score PEMAT score (1-5): 1 being the lowest and 5 being the highest

**Figure 3:**
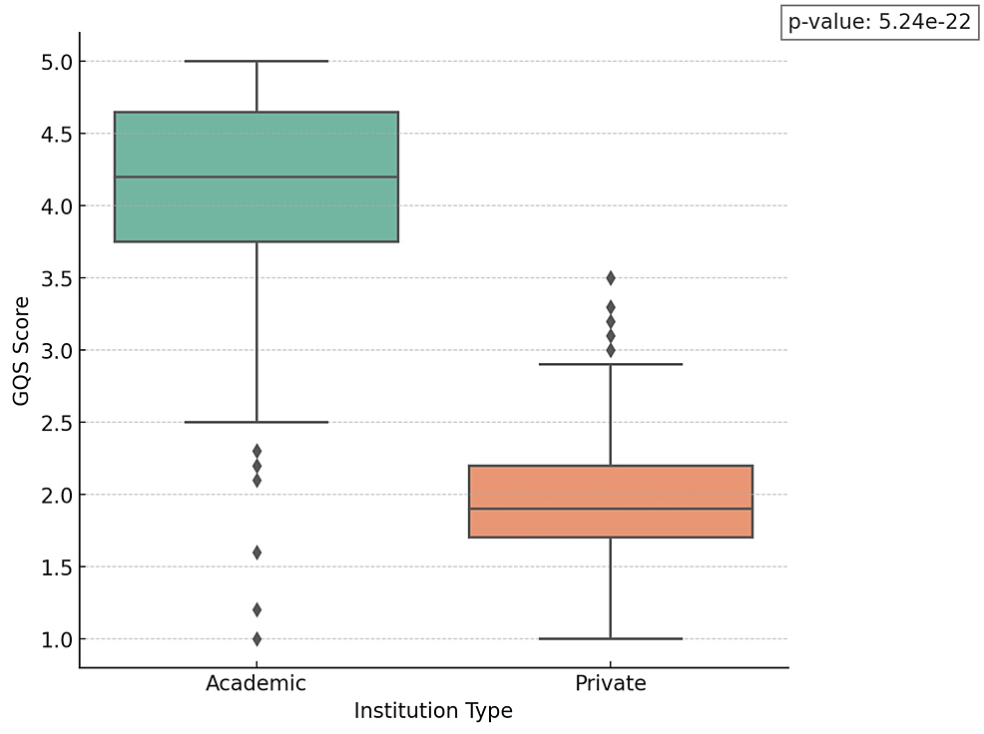
GQS score Global Quality Score (GQS) (1-5): 1 being the lowest and 5 being the highest

Our analysis encompassed 156 YouTube videos, of which 68 (43.5%) originated from academic institutions and 88 (56.5%) from private entities. The evaluation of YouTube content quality, leveraging the DISCERN, PEMAT, and GQS metrics, highlighted notable disparities between videos from academic and private institutions, as shown in Table 2. In every assessed metric, the academic category outperformed the private sector, showing higher average scores: DISCERN (54.61 vs. 34.76, p < 0.01), PEMAT (3.02 vs. 2.11, p < 0.01), and GQS (3.90 vs. 2.02, p < 0.01). These differences are statistically significant, as evidenced by the low p-values, underscoring the reliability of our findings. The small p-values strongly suggest that the discrepancies in scores stem from genuine variations in content quality among the institutions, rather than being merely coincidental. The comparative analysis of the DISCERN, PEMAT, and GQS scores is visually presented in Figures 1, 2, and 3, respectively.

**Table 2:** Top 15 YouTube video review list "M" - represents value in million, "K" - represents value in thousands, DISCERN score (1-80): 1 being the lowest and 80 being the highest, PEMAT score (1-5): 1 being the lowest and 5 being the highest, Global Quality Score (1-5): 1 being the lowest and 5 being the highest

YouTube links	Total number of views	Likes	Dislikes	Subscribers	Publication date	Video duration (min:sec)	Total number of comments	Academic	Private	DISCERN score (0-80)	PEMAT score (1-5)	Global Quality Score (1-5)	Reference
https://www.youtube.com/watch?v=8Zx-uYU1dYM&ab_channel=AmericanCancerSociety	1.16M	18K	309	2.8M	9/5/2017	9:47	534	X		58	3.8	4.1	[11]
https://www.youtube.com/watch?v=y2Bg-spJAG0&ab_channel=RhesusMedicine	1.23M	11K	940	2.44M	10/27/2017	34:22:00	312		X	24	2.1	2.9	[12]
https://www.youtube.com/watch?v=ASv9fhAPmiw&ab_channel=ArmandoHasudungan	146K	220	0	976K	5/20/2022	5:25	Comments turned off	X		55	4	3.5	[13]
https://www.youtube.com/watch?v=CnlT-s0xdHs&ab_channel=MichiganMedicine	54K	1.3K	26	337K	11/29/2021	9:02	38		X	21	2.1	2.5	[14]
https://www.youtube.com/watch?v=C3hwzz29dxY&ab_channel=TODAY	146K	2.3K	234	63.6K	2/22/2019	4:09	0	X		62	3.9	2.2	[15]
https://www.youtube.com/watch?v=asLsuLiPFnY&ab_channel=ABC7NewsBayArea	316K	3K	150	1.5M	8/25/2019	11:44	46		X	25	2.5	2.1	[16]
https://www.youtube.com/watch?v=Ez8ynGn7u2k&ab_channel=WFAA	7.6K	89	125	165K	2/8/2022	14:13	17	X		53	4.2	4	[17]
https://www.youtube.com/watch?v=2pVBJPIMNWQ&ab_channel=CBSNews	30K	647	85	601K	01/26/2019	4:20	31	X		52	3.9	4.1	[18]
https://www.youtube.com/watch?v=MA5XZk_V_TQ&ab_channel=NBCNews	123K	1.2K	147	977K	06/28/2019	19:39	Comments turned off	X		51	3.8	3.7	[19]
https://www.youtube.com/watch?v=2Km-_gHmW3I&ab_channel=NBCNews	867K	13K	152	2.35M	06/20/2013	20:08	741		X	18	2.5	2.1	[20]
https://www.youtube.com/watch?v=PEWt_P1ZIlA&ab_channel=KFYR-TV	160K	2K	74	7.08K	02/10/2021	5:23	178	X		59	4.2	4.5	[21]
https://www.youtube.com/watch?v=8P8aIxudUk8&ab_channel=RoswellParkComprehensiveCancerCenter	241K	3.7K	10	897K	06/22/2022	27:57:00	165		X	24	2.1	1.5	[22]
https://www.youtube.com/watch?v=h_SOfqFJWck&ab_channel=Dana-FarberCancerInstitute	410K	4.9K	25	903K	02/10/2021	7:43	394		X	25	1.3	1.8	[23]
https://www.youtube.com/watch?v=MvH1uEO6GQw&ab_channel=MLive	410K	5K	73	2.35M	02/09/2016	11:45	169		X	22	2.5	2.2	[24]
https://www.youtube.com/watch?v=Pb6fjWaBGPg&ab_channel=ColonCancerCoalition	30.5K	1.1K	157	897K	5/29/2022	2.42	51		X	20	3.1	2	[25]

The findings highlight the critical role of the institutional backdrop in the quality of YouTube's educational offerings. Academic institutions outshine private ones in delivering educational content, likely due to their adherence to stringent content creation standards, comprehensive review processes, and a focused commitment to educational excellence. Such differences underscore the value of academic environments in fostering high-quality educational materials.

This research stresses the importance for viewers to be mindful of the educational content's source on platforms like YouTube, particularly those in pursuit of dependable and superior information. The remarkably low p-values reinforce the credibility of these results, suggesting that the source of educational content whether it originates from an academic or a private institution can significantly influence its quality and reliability.

## Discussion

The power of YouTube in disseminating health information

Accessibility and Reach

Since its founding in 2005, YouTube has grown to be the world's second most visited website, with its content and reach expanding vastly [26]. It garners over two billion daily views, making it a major source of information, particularly on health topics, with 80% of Internet users looking up health information online [27]. However, the minimal content regulation on YouTube and the reliance on personal narratives raise concerns about the accuracy of this information [27]. Studies have illustrated this issue; for instance, negative portrayals of HPV vaccinations in videos increased from 32% in 2008 to 51.7% in 2012 [27]. A 2022 review found only 32% of health-related videos were neutral, highlighting the influence of creator bias [28]. Such statistics urge caution, emphasizing that likes and view counts are not reliable quality indicators [28]. Users should critically assess the health information on YouTube due to the lack of standardized regulations [29].

Educational Content on Colon Cancer Screening

CRC, the second leading cause of cancer-related deaths in the United States, necessitates public awareness [30]. Effective screening and early intervention are key to reducing mortality, but misconceptions pose significant barriers [30]. YouTube, a major information source, impacts public perception of CRC prevention and screening [30]. However, less than half of the related videos discuss protective and risk factors adequately [30]. Studies highlight the importance of emphasizing the risk: the intent for screening fell from 77% to 60% as perceived risk decreased from 5% to 1% [31]. It is also crucial to educate the public about alternative screening methods, such as fecal occult blood tests (FOBT) and fecal immunochemical test (FIT) tests, to enhance the understanding of CRC screening processes [31].

Impact on Public Awareness

Understanding health literacy is crucial for public access to health information, as varying literacy levels affect how people comprehend available information [30]. To enhance the accountability of published videos, especially on platforms like YouTube, measures such as displaying the credentials of educators can be implemented [31]. If organizations and awareness campaigns amplify their presence on such platforms, this could significantly improve the quality and credibility of the information accessible to the public [30]. While healthcare providers cannot control the content their patients consume, it becomes their responsibility to address and rectify any misinformation or misconceptions patients may have encountered [31]. This approach is vital in ensuring that patients receive accurate and reliable health information, ultimately aiding in better health outcomes and informed decision-making [30].

Challenges and perils of YouTube as an information source 

Misinformation and Inaccuracy

YouTube, a key resource for medical education and health information, has become one of the most influential tools on the internet due to its widespread accessibility. However, its reliability is often questioned, as evidenced by a study revealing that 25% of COVID-related information on YouTube was incorrect, complicating the dissemination of accurate knowledge [32]. This issue also pertains to topics like CRC and its screening, where social media platforms can lead to exposure to misinformation and poor-quality content. This can result in unverified health advice, potentially delaying critical care. To address this, quality control measures are being implemented to monitor the health information available to the public. Notably, the World Health Organization is working to establish a new domain dedicated to combating online misinformation, emphasizing strict regulation and accurate information monitoring. This initiative aims to ensure that the public receives reliable and trustworthy health information [33,34].

Quality Control and Regulation

A study has highlighted that YouTube should not be considered a dependable source for health information, as metrics like views and likes are not reliable indicators of content quality. To improve the trustworthiness of health-related information on YouTube, it is suggested that experts review and evaluate the medical content before public release. This approach aims to prevent the dissemination of unverified information, which often leads to misinformation [28]. For instance, research focusing on hepatosteatosis information on YouTube revealed significant variability in content quality, with no clear correlation between the accuracy of information and the number of views or likes. Consequently, the study advises individuals seeking reliable health information, particularly on hepatosteatosis, to focus on the credentials of the content creator rather than popularity metrics like views or likes [35].

The Influence of Non-expert Opinion

Celebrities and influencers exert substantial influence in the digital sphere, often swaying public health decisions through their endorsements or personal health narratives. Angelina Jolie's revelation of her double mastectomy due to the BRCA1 gene notably increased screenings for the gene, highlighting the potential of celebrities to positively impact healthcare choices and reduce disease stigma. However, this influence can also be detrimental, as seen in some celebrities promoting the anti-vaccine movement, undermining efforts and investments in vaccine education, and contradicting scientific evidence [36]. 

The pervasive nature of social media amplifies both positive and negative impacts on public health. For instance, an analysis showed that 81% of foods promoted by musicians at the Teen Choice Awards were high in energy and low in nutritional value, increasing obesity risk among children. These examples underscore the need for a balanced approach to leveraging celebrity influence for health-related matters. Health officials could strategically collaborate with celebrities to promote beneficial health screenings and provide accurate medical advice, harnessing their reach to improve public health outcomes [37].

Comparative analysis

Effectiveness Comparison Between YouTube and Traditional Methods (Brochures and Doctor Consultations)

In the Web 2.0 era, online platforms like YouTube offer a vast array of health information, potentially serving as user-friendly educational tools for CRC patients and their families. While its high traffic and broad reach make it a potent channel for public health campaigns, healthcare providers must be mindful of its limitations in patient education. A meta-analysis revealed that videos and multimedia programs significantly encourage CRC screening, enhancing completion rates [38]. However, a study noted that while YouTube's CRC education videos are high in production quality, their accountability and content scores vary greatly. These videos often miss crucial information needed for effective screening adherence [31]. Unlike outpatient consultations, YouTube lacks interactive communication, preventing patients from addressing specific questions or concerns, and leading to potential knowledge gaps. This highlights the need for a balanced approach to utilizing YouTube as an educational resource in healthcare settings.

Pros and Cons of Each Approach

In a study, the most frequently provided accountability criterion in YouTube videos was the credentials of the main educator, yet only just over half included this information [31]. While credentials lend credibility, most videos lack vital elements like disclosure statements and copyright information. Furthermore, these videos often do not address patients' perspectives, missing discussions on the social impact of disease, screening benefits, and barriers to screening. YouTube content, while abundant, is not always comprehensive or accurate, with some even discouraging routine screenings, negatively influencing preventive health behaviors.

Studies on platforms like TikTok also reveal lower-quality medical content, underscoring the inadequacy of YouTube's popularity metrics, like views and likes, as quality indicators [31]. YouTube should refine its ranking system to prioritize high-quality content. In addition, varying health literacy levels among viewers can impede understanding. Health literacy, defined as the ability to obtain, process, and comprehend basic health information for making informed decisions, is better addressed in doctor-patient consultations, where doctors can tailor advice on CRC screening to individual literacy and knowledge levels [39].

The role of healthcare professionals

Engagement With Social Media

YouTube serves as a valuable tool for healthcare professionals to promote awareness and address misconceptions about colorectal screening. Its capacity to reach a broad audience through audio-visual content, available in various languages and subtitles, makes it an effective platform [40]. The simplicity of the presentation, along with customized feedback and an open comments section, enhances the measurement of a video's impact and reach. For maximum effectiveness, colorectal screening awareness videos should feature credible presenters, utilize both audio and visual aids, and include subtitles, ideally within a concise duration of five to 10 minutes [39]. Engaging video titles and minimal advertisements can further boost viewer engagement, ensuring more people watch the entire video.

Combating Misinformation

Short, impactful colorectal screening videos, lasting less than three minutes, can be strategically used as advertisements preceding popular daily content on YouTube, reaching a vast audience. It is crucial for these videos to directly address the most prevalent questions and stigmas related to colorectal screening, ensuring that they are highly relevant and engaging. Health initiatives for CRC should aim to enhance understanding, alleviate fears and distrust, and promote CRC screening as a vital preventive measure, particularly focusing on older adults [38]. Employing pop-up ads with concise, catchy phrases that tackle common misconceptions or stigmas can significantly boost CRC screening awareness among the general population [40].

Future perspectives

How YouTube Could Evolve to Better Support Accurate Health Information Dissemination

YouTube has launched two key initiatives to enhance healthcare content accessibility for diverse audiences. The first, THE IQ Creator Program, builds on the previous Tackling Health Equity Through Information Quality (THE IQ) initiative, which worked with nonprofits to create health education content [41]. This expansion underscores YouTube's commitment to health equity by empowering content creators [42].

The second initiative is a healthcare-focused pilot program utilizing Aloud, an AI tool developed by Google. It allows creators to dub their videos into various languages, particularly targeting health education. Collaborations with Mass General Brigham, JAMA, Elsevier’s Osmosis, and the Global Health Media Project aim to develop content for Spanish and Portuguese speakers [42].

These YouTube programs represent a strategic effort to address health disparities through improved content accessibility and cultural representation [42]. Leveraging AI and partnerships with renowned health entities, YouTube is enhancing the availability and relevance of health education, empowering diverse communities with accurate and accessible information for better health outcomes [42]. As a major platform, YouTube's initiatives are pivotal in shaping how health information is disseminated globally [42].

Recommendations for Viewers, Content Creators, and Healthcare Professionals

YouTube is intensifying its efforts to combat medical misinformation by evaluating videos under its specific policies, aligning with public health risks and authoritative health guidance [43]. The platform's assessment will target three key misinformation categories: prevention, treatment, and denial [43]. In the realm of prevention misinformation, YouTube will scrutinize content that contradicts established health recommendations, including misleading information on vaccines and illness prevention methods [43]. Treatment misinformation includes videos advocating for unscientific medical practices or discouraging proven treatments [43]. Lastly, denial misinformation focuses on content falsely denying the existence or severity of medical conditions, like incorrect claims about COVID-19 fatalities [43]. Through this structured approach, YouTube aims to enhance public health awareness and ensure viewers have access to accurate and reliable medical information [43].

Expert-Driven

Evaluations play a crucial role in establishing the credibility of health websites, with quality certifications often based on expert assessments [29]. However, given the overwhelming volume of online content (72 hours of video uploaded every minute), relying solely on expert reviews is impractical [29]. A more sustainable approach is to harness social networking dynamics [41]. Utilizing collective intelligence, such as peer reviews from online communities and social network metrics, can effectively gauge content trustworthiness [44]. Encouraging active participation from health consumers and producers to endorse or flag content not only improves visibility but also helps in filtering out misleading information, making the online health information landscape more reliable and informative [45].

Popularity-Driven

This manuscript examines the use of popularity metrics, such as view counts and public ratings, as indicators of content quality on YouTube [14]. It points out that these quantitative measures can be easily manipulated through marketing strategies or viral trends, leading to potentially misleading assessments of content quality [29]. The paper also discusses how online crowd influence can significantly impact consumer health decisions, with a notable 28.5% chance of decision alteration following social feedback [46]. It raises concerns about the reliability of public ratings and flags, highlighting instances of popular but inappropriate videos [29]. The study suggests adopting guidelines, like those of the CDC, for managing risks in viral scenarios, given YouTube's role as a major opinion-sharing platform [47]. In addition, it underscores the negative effects of unsolicited comments on public health campaigns, exemplified by the changing discourse on HPV vaccination on YouTube over time [48,49].

Heuristic-Driven

Researchers often rely on video metadata like length to gauge quality, but this approach lacks solid evidence-based reasoning [29]. These metrics should be regarded as indicators of potential viewer interest, rather than direct measures of quality [29]. There is a risk that high-quality content may be overlooked due to inadequate metadata, while videos with misleading information but fitting metadata might be mistakenly deemed high quality [29]. The study underlines the importance of considering both content and metadata for a more accurate assessment of video quality, cautioning against the pitfalls of focusing solely on one aspect [29].

Given the ever-increasing volume of YouTube content, a more effective evaluation strategy would involve a multi-dimensional approach. This would combine a social network perspective with criteria established by various experts, including laypersons, professionals, and organizations, alongside heuristic indicators [29]. Such a comprehensive method promises to more effectively manage the massive content growth on YouTube, ensuring a thorough and accurate assessment of video quality [29].

This study acknowledges several limitations that may impact the interpretation and generalizability of its findings. First, the research focused exclusively on English-language videos, potentially excluding relevant content in other languages that could offer different perspectives on CRC screening awareness. In addition, the assessment of video content quality relied on specific evaluation tools (DISCERN, GQS, and PEMAT), which, although validated, may not capture all aspects of educational effectiveness or viewer engagement. The study's methodology, which involves the subjective interpretation of video content by researchers, also introduces the possibility of bias. Moreover, the dynamic nature of YouTube content, where videos are constantly uploaded, updated, or removed, means that the analysis may not represent the platform's current state. Lastly, the reliance on public metrics like views and likes as indicators of content reach and acceptance does not necessarily reflect the educational impact or accuracy of the information presented, limiting the ability to fully gauge the effectiveness of CRC screening awareness efforts on YouTube.

## Conclusions

This study presents a compelling insight into the realm of YouTube as an educational platform for CRC awareness, underscoring the significant disparities in content quality between academic and private institutions. The rigorous statistical analysis highlights the superior quality of academic content, which is attributed to stringent content creation standards and a focused commitment to educational excellence. These findings emphasize the critical importance of source credibility and the potential impact of misinformation on public health outcomes. As YouTube continues to be a pivotal source of health information, the necessity for viewers to critically evaluate content sources and for content creators to uphold high-quality standards becomes increasingly apparent. This research not only sheds light on the current state of CRC educational content on YouTube but also calls for enhanced regulatory oversight and the promotion of accurate, reliable health information to better serve the public's need for trustworthy medical guidance.
